# Structure and intermolecular interactions of the rare amide–pyridine synthon: a cocrystal of nicotinamide and 2-chloro-3-hydroxypyridine

**DOI:** 10.1107/S2053229626004882

**Published:** 2026-05-18

**Authors:** Oluwatoyin Akerele, Andreas Lemmerer

**Affiliations:** a Jan Boeyens Structural Chemistry Laboratory, Molecular Sciences Institute, School of Chemistry, University of the Witwatersrand, Private Bag 3, PO Wits, 2050, Johannesburg, South Africa; University of Oxford, United Kingdom

**Keywords:** crystal structure, inter­molecular inter­actions, co­crys­tal, 2-chloro­pyridin-3-ol, nicotinamide

## Abstract

Nicotinamide (Nico) and derivatives of pyridine are important materials in both the pharmaceutical and agrochemical industries. In the 21st century, pyridine-based agrochemical products achieved commercial success because of their structural diversity and different modes of action that can be explored to improve the effectiveness of the com­pounds. In this article, we explore the co­crys­tallization of nicotinamide/isonicotinamide and substituted pyridines and their synthons to understand their ease of formation. A co­crys­tal of Nico and 2-chloro-3-hy­droxy­pyridine (2Cl3OHPY) was synthesized using solution and mechanochemical methods, and characterized by X-ray diffraction. The structural stability and inter­molecular inter­action of the (Nico)·(2Cl3OHPY) co­crys­tal were investigated using differential scanning calorimetry (DSC) and density functional theory (DFT). The co­crys­tal has strong chain (N—H⋯N), dimer (N—H⋯O) and discrete (N—H⋯O) hy­dro­gen bonds with energy strengths of −31.21, −66.99 and −36.82 kJ mol^−1^, respectively, and a short C—H⋯π bond that builds a twisted three-dimensional structure (viewed along the *c* axis) and stabilizes the crystal packing. The results show that the com­pound is chemically stable, and the two dominating inter­actions are electrostatic and dispersion energies. An analysis of the aromatic amide and pyridine synthon in the CSD reveals the presence of close supporting inter­actions that strengthen the N—H⋯N hy­dro­gen bond. The understanding of the structural properties and inter­molecular inter­actions in the (Nico)·(2Cl3OHPY) co­crys­tal and NH_2_⋯N_py_ synthon provided in this study could be used to design materials for different applications, including pigments, explosives, drugs, agrochemicals and food additives.

## Abstract 2

Considering its low occurrence in the Cambridge Structural Database, is the amide–pyridyl synthon easy to form, and what structural factors affects its inter­action energy and its subsequent formation?

## Introduction

Cocrystallization is a technique that is used in different fields, such as pharmaceuticals, agrochemicals and materials science, to improve the properties of com­pounds and design materials with desired properties (Dutt *et al.*, 2021 ▸). The strategy for designing co­crys­tals incorporates the chemical identity of the com­ponents, the inter­molecular inter­actions and the crystallization method. A co­crys­tal is a multi-com­ponent crystal containing two or more neutral mol­ecules in a definite stoichiometric ratio; it is formed primarily through strong hy­dro­gen-bonding inter­actions and has a unique structure and properties com­pared to the single com­ponents (Bond, 2011 ▸). Amide and pyridine functional groups are commonly found in many pharmaceutical and agrochemical active ingredients (Mohabbat *et al.*, 2024 ▸; Ling *et al.*, 2021 ▸; Diniz *et al.*, 2018 ▸). In fact, pyridine-based agrochemical products have achieved commercial success in the 21st century because of their structural diversity and different modes of action that can be explored to improve the effectiveness of the com­pounds (Zakharychev & Martsynkevich, 2024 ▸; Wang *et al.*, 2025 ▸; Guan *et al.*, 2016 ▸). These pyridyl mol­ecules also co­crys­tallize with other com­pounds through noncovalent inter­actions to form co­crys­tals or salts. However, the amide–pyridine synthon has the least probability of occurrence in the Cambridge Structural Database (CSD; Groom *et al.*, 2016 ▸) when com­pared with both homosynthons and heterosynthons of acid, amide and pyridine functional groups (Babu *et al.*, 2007 ▸). In fact, a search of the CSD in June 2025 revealed the number of amide and pyridyl synthons to be less than 10% of carb­oxy­lic acid and pyridyl synthons (Bruno *et al.*, 2002 ▸).
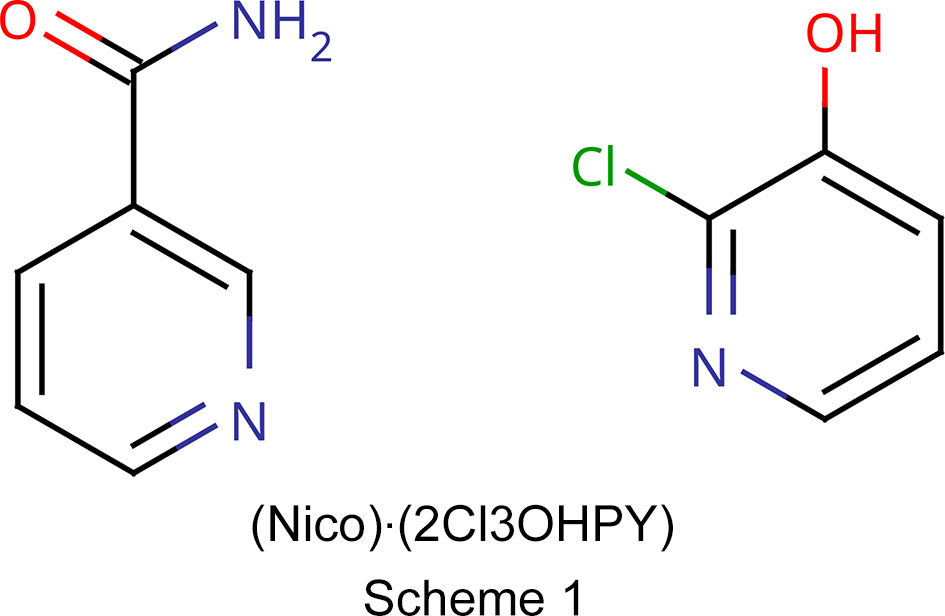


Similarly, a specific search for nicotinamide or isonicotinamide and pyridine derivatives yields less than 20 hits in the CSD. Studies have analyzed the synthons and inter­actions between OH⋯N_py_ and COOH⋯N_py_ (Lemmerer *et al.*, 2013 ▸; Bis *et al.*, 2007 ▸; Shattock *et al.*, 2008 ▸; Ganie *et al.*, 2022 ▸; Goswami *et al.*, 2016 ▸; Kavuru *et al.*, 2010 ▸; Sharma *et al.*, 2022 ▸; Ngoma Tchibouanga & Jacobs, 2020 ▸; Sowmya & Kumar, 2023 ▸; Jarzembska *et al.*, 2017 ▸; Kusuma *et al.*, 2022 ▸), but there is no study, to the best of our knowledge, that has examined the NH_2_⋯N_py_ synthon, its structure and inter­actions. On this note, we conducted a systematic set of co­crys­tallization experiments on nicotinamide/isonicotinamide and a series of pyridine derivatives using both solution and mechanochemical methods to understand the ease of formation, structure and inter­molecular inter­actions of the multi-com­ponent crystals. There was, however, only one success, and so this study has focused on the analysis of the structure and inter­molecular inter­actions of the co­crys­tal formed by nicotinamide (Nico) and 2-chloro-3-hy­droxy­pyridine (2Cl3OHPY) (Scheme 1), as well as the related structures (NH_2_⋯N_py_ synthon) in the CSD. The pattern revealed in this study could be used to design novel materials and influence the building of a predefined crystal network.

## Experimental

### *Conquest* search

**Figure 1:**
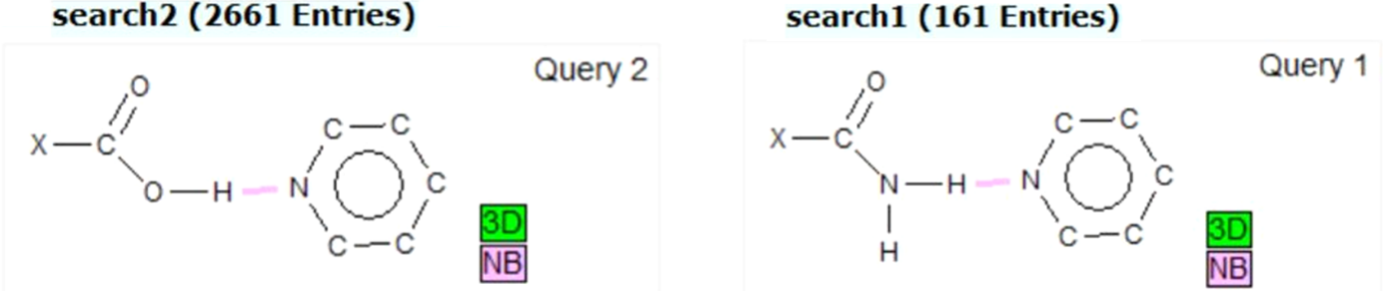
The query fragment for the synthon search.

A search for carb­oxy­lic acid and the pyridyl ring, including a H⋯N contact, in the CSD, with filters for 3D coordinates determined and only organics, gave 2661 hits. The search for amide and the pyridyl ring with the same filters gave only 161 hits, which is less than 10% of the former, as shown in Fig. 1 ▸.

The average hy­dro­gen-bond distance in COOH⋯N_py_ is much lower than in CONH_2_⋯N_py_, as shown in Table 1 ▸. This suggests that the hy­dro­gen-bond strength of COOH⋯N_py_ is stronger than the CONH_2_⋯N_py_ hy­dro­gen bond, which cor­relates with the literature. The strength of the inter­action energy of the amide–pyridine synthons is one of the factors contributing to their low occurrence in the CSD. Since there is a connection between the mol­ecular structure and the crystal structure formed (Nangia & Desiraju, 1999 ▸), analysis of the structure and inter­actions (both strong and weak bonds) of the new co­crys­tal and related architecture will provide an insight into the factors that control the crystal packing.

Analysis of the 161 hits (amide and pyridyl synthon) reviews the proportion for which the NH_2_⋯N_py_ hy­dro­gen bonds occur in the CSD: 50 hits are seen in a com­ponent crystal that has both pyridyl ring and amide functional groups (for example, refcode LOCSOP; Dyachenko *et al.*, 2023 ▸), 62 hits occur between nicotinamide or isonicotinamide in a com­ponent and multi-com­ponent environments [KIPGUO (Surov *et al.*, 2022 ▸) and JEDHOT (Li *et al.*, 2018 ▸)] and 49 are found between two com­ponents of pyridyl and amide mol­ecules. There is an even distribution among these groups, which suggests that the amide–pyridyl hy­dro­gen bond will form irrespective of its environment. An analysis of the subset of 49 hits shows that 70% were between aliphatic amides (formamide and urea) and pyridyl mol­ecules, while 30% were between aromatic amides and pyridyl mol­ecules.

#### Analysis of the aromatic NH_2_⋯N_py_ synthon and structures in the CSD

– The crystal structures with aromatic amide–pyridyl synthons along with their bond angles and inter­action energies are shown in Table 2 ▸. The average inter­action energy (calculated using *GAUSSIAN16*, B3LYP/def2-TZVP; Frisch *et al.*, 2016 ▸) of the NH_2_⋯N_py_ bond is −33.26 kJ mol^−1^.

– The bond angles do not correlate with the inter­action energy; for instance, com­pounds with carbonyl groups at positions 2 and 6 of a pyridyl ring has low angles but strong inter­action energies (see structures I–III). Conversely, bulky com­pounds with two or more phenyl rings have high angles but weak inter­action energy (see structures VIII, IX, XI and XII). However, it should be noted that structures IV–VII show correlation between the bond angle and inter­action energy. Therefore, the bond angles should be used with care to determine the strength of the bond between the aromatic amide and pyridyl co­crys­tallization components.

– Analysis of the structure and inter­actions of aromatic amide–pyridyl synthons reveals the functional groups and additional supporting inter­actions closer to the NH_2_⋯N_py_ bond as factors influencing the strength of the bond. For instance, the presence of carb­oxy­lic acid groups at positions 2 and 5 of pyridine strengthens the NH_2_⋯N_py_ bond, as seen in structures I–III. However, there is approximately a −13 kJ mol^−1^ reduction in energy when there is only one carb­oxy­lic group at position 2 or 5, as seen in structures II and III. Structures IV and V have a nitro group at positions 3 and 5 of the aromatic amides and the supporting inter­action (C—H⋯N) contributes to the strength of the NH_2_⋯N_py_ bond. On the other hand, with the presence of hydroxyl and fluorine groups at positions 3 and 5 of the aromatic amide, the bonds formed in structures VI and VII do not improve the strength of the NH_2_⋯N_py_ bond. Similarly, a C—H⋯π supporting inter­action in structures VIII–IX does not lead to an appreciable increase in the inter­action energy of the NH_2_⋯N_py_ bond. The inter­action energy of the NH_2_⋯N_py_ bond in structures X–XII is quite low; this could be due to the absence of a strong supporting inter­action closer to the NH_2_⋯N_py_ bond.

– It should be noted that these observations are drawn from a small set of aromatic amide–pyridyl synthons and other factors beyond the inter­est of this study could be contributing to its low occurrence in the CSD.

### Synthesis

The selected chemicals in Table 3 ▸ were purchased from Sigma–Aldrich and used without further purification as received. The crystallizations were carried out using the two common crystal growth methods: slow (solvent) evaporation and mechanochemical.

#### Slow evaporation method

20 mg of nicotinamide or isonicotinamide was dissolved in 1.5 ml of ethanol and an appropriate molar ratio of the substituted pyridine was added slowly to the prepared solution to give a 1:1 molar ratio and concentrations of 0.11 *M*. The solution was heated and stirred gently on a hotplate at a low tem­per­a­ture of about 30 °C until the com­pound dissolved com­pletely. The heated solution was cooled to room tem­per­a­ture before the vial was covered with a perforated Parafilm sheet to allow for the evaporation of the solvents. Crystals formed within 5 d and the morphology of the crystals was examined visually under a microscope. A suitably sized crystal of the com­plex was carefully selected for single-crystal X-ray diffraction (SC-XRD) to ascertain the formation of a multi-com­ponent crystal. Powder X-ray diffraction (PXRD) and differential scanning calorimetry (DSC) were also used to analyze the crystals.

#### Mechanochemical method

80 mg of nicotinamide/isonicotinamide and the equivalent 1:1 molar ratio of the substituted pyridine were weighed into a 1.5 ml plastic vial with one stainless steel ball of 4 mm diameter and 10 drops of ethanol. The vials were placed in an adapter for six reactions, and the slurry grinding experiment was carried out in a Retsch MM 400 Mixer Mill at room tem­per­a­ture. The loaded vials were shaken in the Mixer Mill for 60 min at 25 rpm^−1^ or Hz frequency. PXRD analysis was conducted after the resulting powder was dried at 50 °C in an oven. However, there was no new phase formation in all the ground crystallization experiments except for the (Nico)·(2Cl3OHPY) co­crys­tal. The PXRD results for the unsuccessful co­crys­tals with the starting materials are given in the supporting information.

### Refinement

Crystal data, data collection and structure refinement details are summarized in Table 4 ▸. All atoms were refined anisotropically before the inclusion of H atoms. H atoms on aromatic rings were placed in calculated positions, while the *sp*^3^-hybridized C atoms were derived from electron-density maps. The H atom on the N atom was also derived from an electron-density difference map and refined freely. All images, including the crystal packing, were created using *Mercury* (Macrae *et al.*, 2020 ▸).

### Powder X-ray diffraction (PXRD)

**Figure 2:**
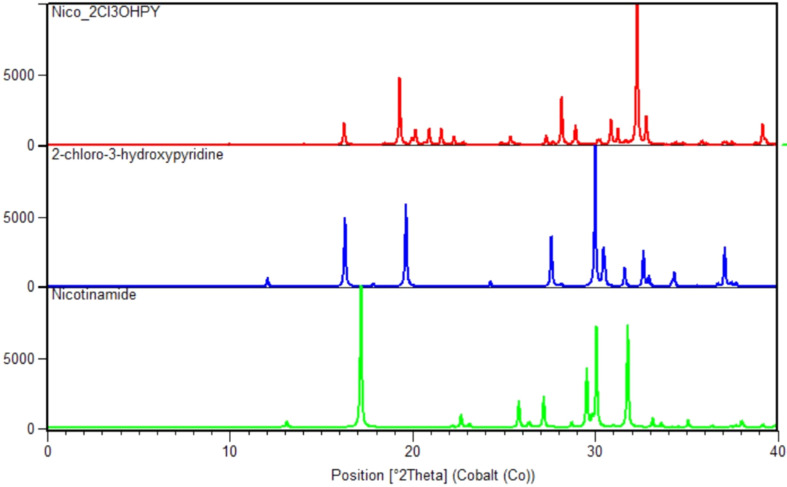
Simulated PXRD patterns: blue is 2-chloro-3-hy­droxy­pyridine, green is nicotinamide and red is the new multi-com­ponent co­crys­tal (Nico)·(2Cl3OHPY).

PXRD was used to further confirm the formation of the new co­crys­tal. A diffractogram of a powdered crystalline sample of the multi-com­ponent crystal was measured at 293 K using a Bruker D2 phaser powder X-ray diffractometer. The instrument is equipped with a sealed tube Co *K*α_1_X-ray source (λ = 1.78896 Å) and a LynxEye PSD detector in Bragg–Brentano geometry, and operating at 30 kV and 10 mA. The data collection was carried out with a scanning inter­val ranging from 2θ = 5.0015 to 40° at a scan speed of 0.5 s per step (with an increment step size of 0.028445°). The experimental and simulated powder pattern of the new co­crys­tal is given in the supporting information. The overlay of the simulated patterns pre­sent­ed in Fig. 2 ▸ shows the different intensity peaks for nicotinamide, 2-chloro-3-hy­droxy­pyridine and the (Nico)·(2Cl3OHPY) co­crys­tal.

### Thermal analysis

**Figure 3:**
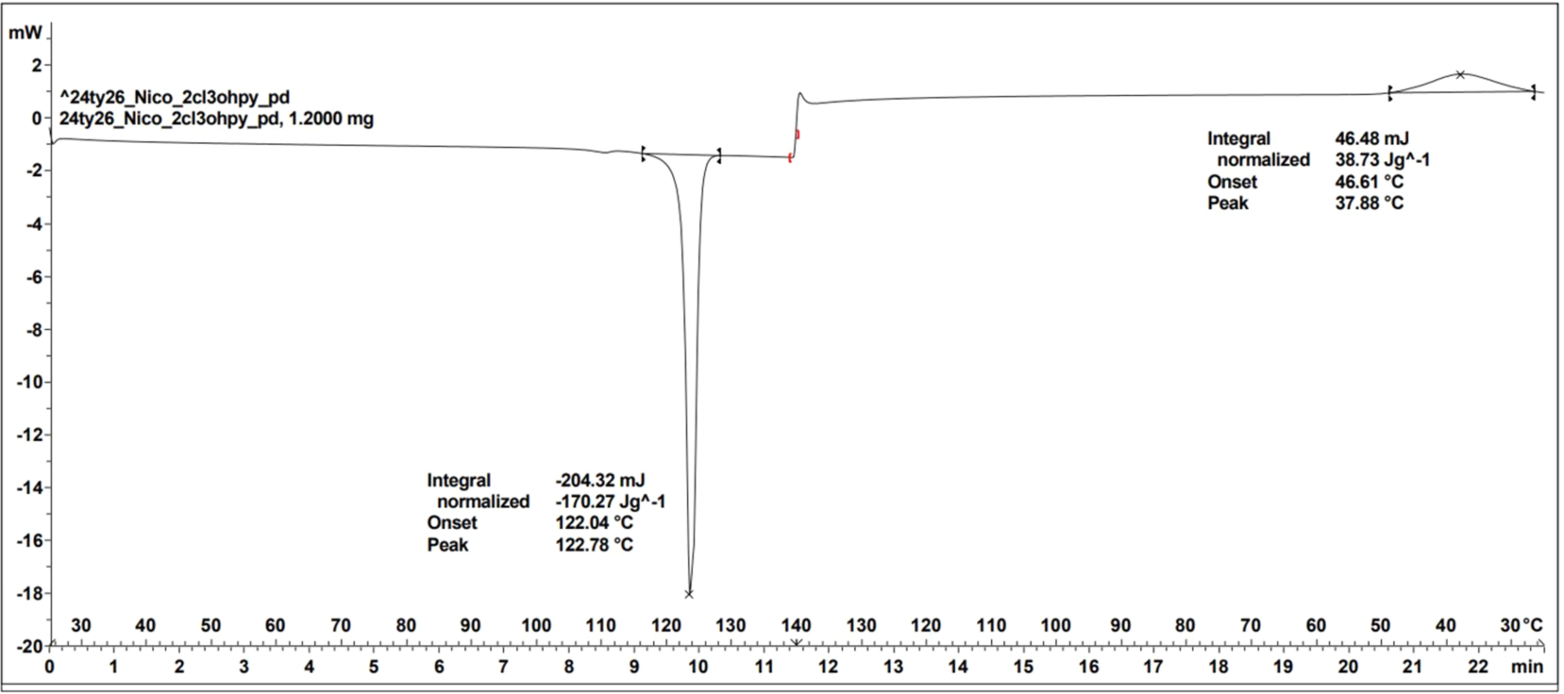
The DSC scan of (Nico)·(2Cl3OHPY).

Differential scanning calorimetry (DSC) was used to measure the change in the heat flow of the sample as tem­per­a­ture changes (Newman & Wenslow, 2018 ▸). The sample was heated and cooled to determine the melting points, enthalpies of phase transitions and stability of any different phases. The tem­per­a­ture and energy calibrations were performed using pure indium (purity 99.99%, m.p. 156.6 °C, heat of fusion 28.45 J g^−1^) and pure zinc (purity 99.99%, m.p. 419.5 °C, heat of fusion 112 J g^−1^). A Mettler Toledo DSC 3 was used to collect the DSC data and aluminium pans were placed under nitro­gen gas at a flow rate of 10 ml min^−1^. At a heating or cooling rate of 10 °C min^−1^, the samples were heated from 25 °C to the final tem­per­a­ture, which is the tem­per­a­ture just after the sample melting point, as visually established from the hot stage, and then cooled to 25 °C. The exothermic peak, which is the amount of heat (energy) released as the tem­per­a­ture changes, was used to determine the thermal stability of the multi-com­ponent crystal. The plot of the DSC thermogram for the (Nico)·(2Cl3OHPY) is given in Fig. 3 ▸.

### Computational studies

#### Inter­action energy between mol­ecules in units in the multi-com­ponent crystal

The *GAUSSIAN16* suite of programs was used for the optimization of the H-atom positions with the default Berny algorithm (Frisch *et al.*, 2016 ▸; Li & Frisch, 2006 ▸). The H-atom positions of the mol­ecular structure obtained from the crystal structures were optimized in the gas phase at the B3LYP functional with the def2-TZVP basis set and incorporate Grimme’s D3 dispersion correction for a proper description of the dispersion inter­actions (Becke, 1997 ▸; Becke, 1992 ▸; Zhao & Truhlar, 2008 ▸; Grimme *et al.*, 2010 ▸). The H-atom positions from crystal structures are not accurately determined by X-ray crystallography. This was done to obtain the inter­action energy within the two mol­ecules in a unit and was calculated with the same theoretical method (B3LYP-D3/def2-TZVP), taking the basis set superposition error (BSSE) into account with the counterpoise correction (Simon *et al.*, 1996 ▸; Boys & Bernardi, 1970 ▸; Ransil, 1961 ▸). The theoretical details can be found in our previous article (Akerele & Lemmerer, 2025 ▸).

*Chemcraft* (Zhurko, 2026 ▸) software was used to analyze and visualize the output generated from *GAUSSIAN16* calculations.

#### Hirshfeld surfaces and inter­molecular inter­actions

*CrystalExplorer* (Spackman *et al.*, 2021 ▸) was used to generate the Hirshfeld surfaces (HS) at high standard resolution using the CIF as the input file. *CrystalExplorer* creates colour-coded and HS surface maps that help to visualize the important regions of the inter­molecular inter­actions on the surface. The standard normalized contact (*d*_norm_) of Hirshfeld surface analysis is given as follows:
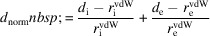
where *d*_i_ is the distance that represents the nucleus inside the surface and *d*_e_ is the distance from the HS to the nearest core outside the surface (Dege *et al.*, 2022 ▸).

The *d*_norm_ is the range of distances between the surface and the nearest atomic external surfaces (*d*_e_) and inter­nal surfaces (*d*_i_). Red contacts are those that are shorter than the van der Waals radii (vdW), indicating that the atoms that form inter­molecular bonds are closer than the sum of their radii (Garg & Azim, 2022 ▸). Contacts with distances equal to the sum of the van der Waals radii are shown on the white surface. A blue colour indicates inter­actions that are more distinct – that is, contacts that are longer than the sum of the van der Waals radii (Dege *et al.*, 2022 ▸; Zeng *et al.*, 2023 ▸; Garg & Azim, 2022 ▸; Garg *et al.*, 2021 ▸; Garg *et al.*, 2022 ▸).

*CrystalExplorer* was also used to calculate the lattice energies of the mol­ecular systems. The wavefunctions of the mol­ecular system were calculated with the built-in *TONTO* program at the CE-B3LYP/6-31G(d,p) theoretical level (Jayatilaka & Grimwood, 2003 ▸; Mackenzie *et al.*, 2017 ▸; Turner *et al.*, 2015 ▸). All the energies of inter­action between the selected mol­ecule (at the centre of the cluster) and its neighbouring mol­ecules were com­puted; the model then separated the total energies into different com­ponents, such as electrostatic, polarization, dispersion and repulsion energy com­ponents (Spackman *et al.*, 2008 ▸).

## Results and discussion

### Crystallization experiment

The crystallization experiment results between nicotinamide or isonicotinamide and all the substituted pyridines listed in Table 2 ▸ were unsuccessful, except for nicotinamide (Nico) and 2-chloro-3-hy­droxy­pyridine (2Cl3OHPY), which gave a co­crys­tal *via* both crystallization methods. This is the only multi-com­ponent co­crys­tal obtained and was discovered for the first time in this study. The unsuccessful crystallization experiments led to the formation of crystals of each starting material only.

### Mol­ecular structure of (Nico)·(2Cl3OHPY)

**Figure 4:**
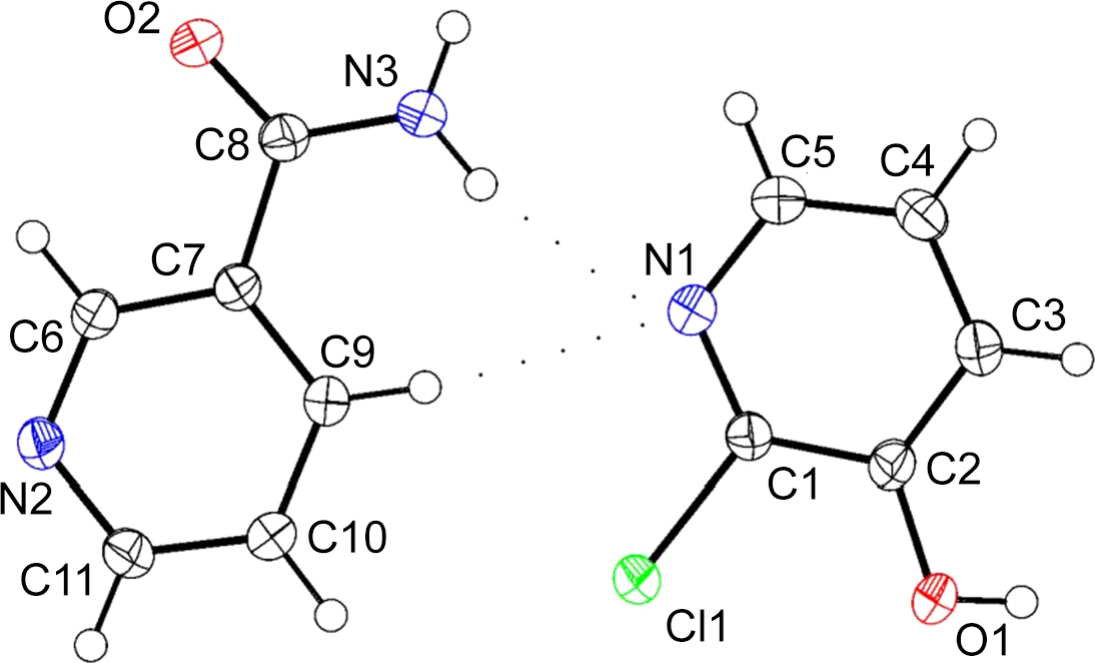
The mol­ecular structure of the co­crys­tal, showing the atom-numbering scheme of the asymmetric unit. Displacement ellipsoids are drawn at the 50% probability level and H atoms are shown as small spheres of arbitrary radii.

The asymmetric unit of multi-com­ponent crystal (Nico)·(2Cl3OHPY) is shown in Fig. 4 ▸.

#### Crystal packing of the mol­ecular structure

**Figure 5:**
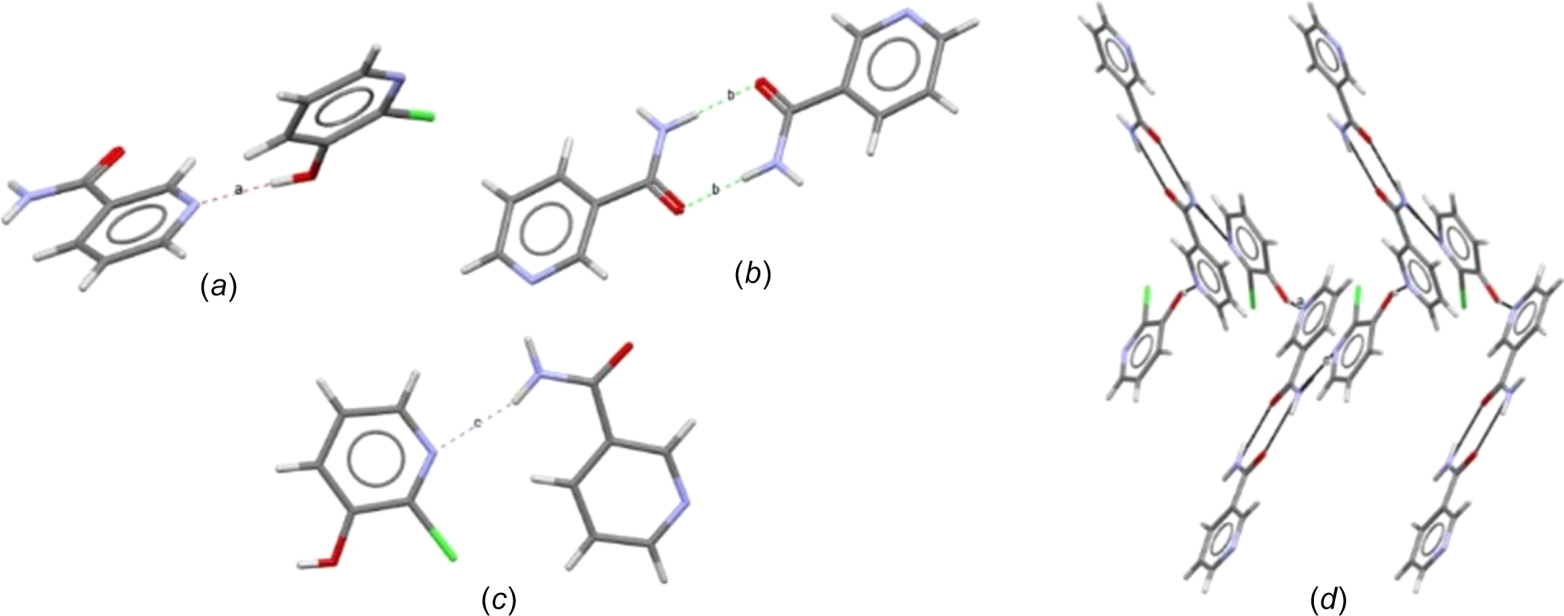
(*a*) The discrete *D*(2) hy­dro­gen bond, (*b*) the dimer 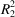
(8) hy­dro­gen bond, (*c*) the chain *C*(2) hy­dro­gen bond and (*d*) the mol­ecules packed in opposite directions in a spiral-like manner along the *c* axis.

(Nico)·(2Cl3OHPY) crystallized in the monoclinic space group *P*2_1_/*n* with *Z*′ = 2. Fig. 5 ▸ shows the units that drive the packing arrangement of the mol­ecular structure. The asymmetric unit has two symmetry-independent mol­ecules with a discrete *D*(2) hy­dro­gen bond (Bernstein *et al.*, 1995 ▸), through O1—H1⋯N2 [Fig. 5 ▸(*a*)]. Two mol­ecules of nicotinamide related by an inversion operation form a dimer hy­dro­gen bonded with a 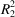
(8) motif through N3—H3*A*⋯O2^i^ [Fig. 5 ▸(*b*)]. The mol­ecules in Fig. 5 ▸(*c*) form a chain of hy­dro­gen bonds through N3—H3*B*⋯N1^ii^. The asymmetric unit bonds with the nicotinamide through dimer hy­dro­gen bonds in one direction and 2Cl3OHPY through a chain hy­dro­gen bond in another direction in a spiral-like manner along the *c* axis, as shown in Fig. 5 ▸(*d*).

**Figure 6:**
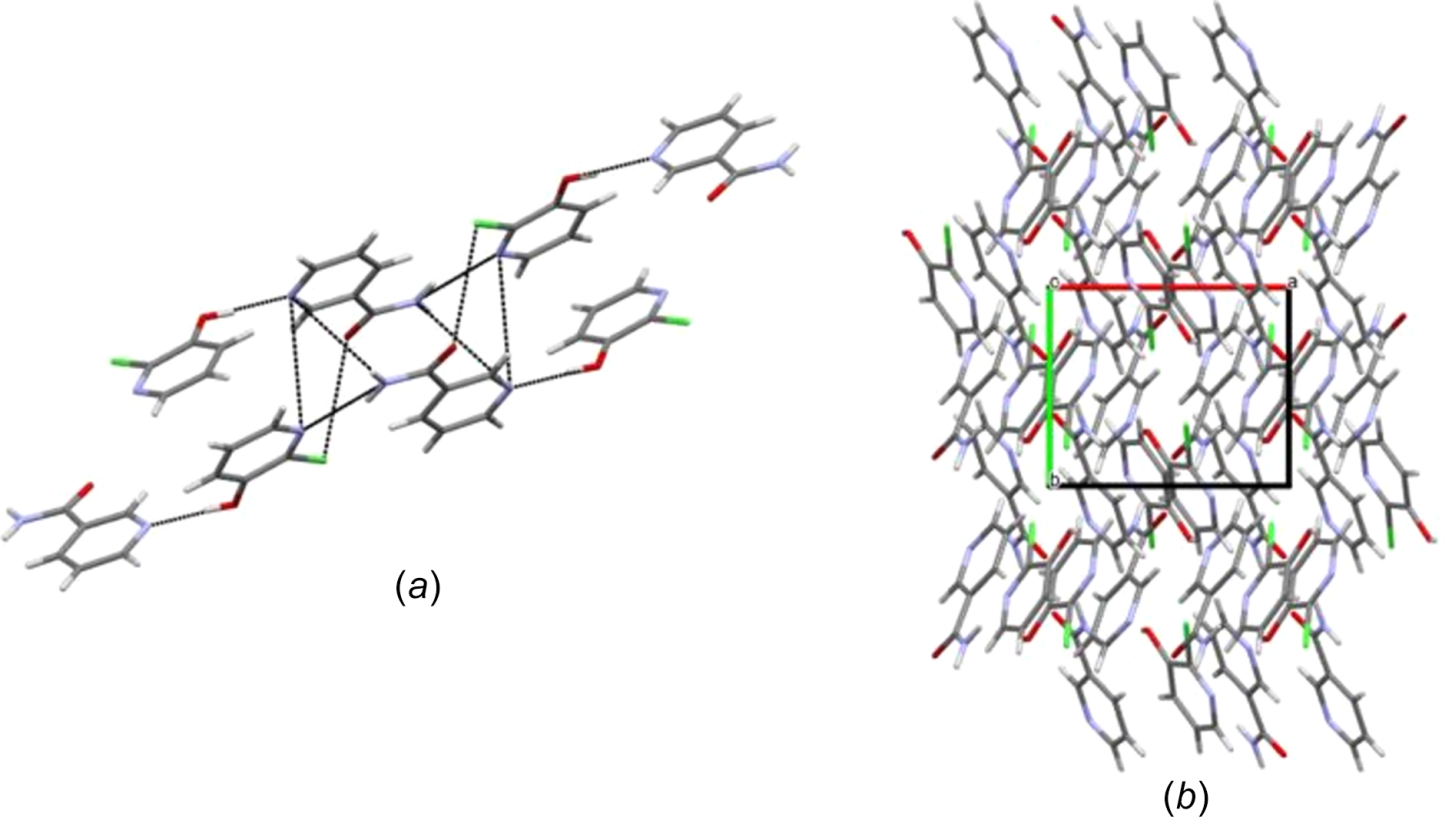
(*a*) The noncovalent inter­actions joining adjacent chains [symmetry code: (i) *x* + 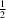, −*y* + 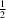, *z* − 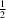]. (*b*) The overall packing arrangement along the *b* axis.

The asymmetric units also stack anti­parallel and adjacent to one another through short contacts between N3—H3*A*⋯N2, N3—H3*B*⋯N1, N1⋯N2 and Cl1⋯O2, as shown in Fig. 6 ▸(*a*), which resulted in the overall twisted packing arrangement along the *c* axis, as listed in Fig. 6 ▸(*b*).

#### Inter­molecular hy­dro­gen bonds in the (Nico)·(2Cl3OHPY) co­crys­tal

The hy­dro­gen bonds between the two starting com­ponents are shown in Table 5 ▸.

### Theoretical studies

#### Energetic properties of the (Nico)·(2Cl3OHPY) structure

The energy of inter­action within the asymmetric unit and other units are −31.21, −66.99 and −36.82 kJ mol^−1^ for Figs. 5 ▸(*a*), 5(*b*) and 5(*c*), respectively, in the gas phase. The strength of the hy­dro­gen bonds of all three units is strong and contributes to the overall stability of the crystal structure. The N—H⋯O hy­dro­gen-bonded dimer [Fig. 5 ▸(*b*)] contributes −33.50 kJ mol^−1^ per donor, which falls within the discrete and chain hy­dro­gen-bond energy.

A −5 kJ mol^−1^ increase is observed in the inter­action energy of Figs. 5 ▸(*a*) and 5(*c*); this further indicates that the strength of inter­action of O—H⋯N is stronger than N—H⋯N. This suggests that the strength of energy (hy­dro­gen bond) could be one of the factors that is not favouring the formation of the N—H⋯N hy­dro­gen bond, alongside the elusive crystallization of nicotinamide/isonicotinamide and substituted pyridine co­crys­tal, thereby leading to the lower number of amide–pyridyl structures in the CSD.

#### Hirshfeld surface (HS) analysis of the (Nico)·(2Cl3OHPY) co­crys­tal

To visualize the mol­ecular packing and inter­actions in the crystal structures, a HS analysis was carried out using *CrystalExplorer* (Version 21) (Dege *et al.*, 2022 ▸; Spackman & Jayatilaka, 2009 ▸).

The HSs and fingerprint plots (FPs) are given in the supporting information (Figs. S7–S11). The HSs show intense red regions for N—H⋯O hy­dro­gen bonds and light-red regions for C⋯O and H⋯H contacts. The FPs show high percentages for the following inter­actions: H⋯H 24.5%, O⋯H/H⋯O 18.0%, Cl⋯H/H⋯Cl 16.2%, C⋯H/H⋯C 15.0% and N⋯H/H⋯N 10.6%. These results confirm the importance of these inter­actions in the (Nico)·(2Cl3OHPY) co­crys­tal.

#### Inter­action energy of the (Nico)·(2Cl3OHPY) crystal structure

The addition of inter­action energy calculations in *CrystalExplorer* allows for the precise calculation of the intensity of inter­actions, which may be directly com­pared to the outcomes obtained from HS analysis.

The CE-B3LYP/6-31G(d,p) energy model, which is accessible in *CrystalExplorer21* (Turner *et al.*, 2017 ▸; Garg *et al.*, 2022 ▸; Akhileshwari *et al.*, 2022 ▸; Frisch *et al.*, 1984 ▸), is used to com­pute the lattice energy. A cluster of mol­ecules is created by applying crystallographic symmetry operations to a chosen central mol­ecule within a radius of 20 Å (Hirshfeld, 1977 ▸). The total energies (*E*_tot_) are separated into different com­ponents, such as electrostatic (*E*_ele_), polarization (*E*_pol_), dispersion (*E*_dis_) and repulsion (*E*_rep_) energies (Chen *et al.*, 2018 ▸), with respective scale factors of 1.057, 0.740, 0.871 and 0.618.

The summation of the lattice energy (in kJ mol^−1^) for the (Nico)·(2Cl3OHPY) structure is −66.750 (*E*_ele_), −14.097 (*E*_pol_), −64.650 (*E*_dis_), 58.138 (*E*_rep_) and −87.358 kJ mol^−1^ (*E*_tot_) for N—H⋯O. The evaluation of the energy com­ponents shows that the electrostatic and dispersion energies are the highest contributors to the stability of the structure. This suggests that hy­dro­gen bonds and other noncovalent inter­actions are contributing to the stability of the (Nico)·(2Cl3OHPY) crystal structure.

## Discussion

The present study combines a series of substituted pyridines with nicotinamide and isonicotinamide using two common crystal growth methods; however, only one co­crys­tal was obtained. The crystal structure, (Nico)·(2Cl3OHPY), has strong O—H⋯N, N—H⋯O and N—H⋯N hy­dro­gen bonds, and weak noncovalent inter­actions that stabilize and drive the twisted three-dimensional packing arrangement along the *c* axis. The inter­action energy of the NH_2_⋯N_py_ hy­dro­gen bond is −31.21 kJ mol^−1^, and is supported by a close inter­action between the C—H⋯N hydrogen bond. The FP and HSs confirm the importance of these inter­actions (O⋯H/H⋯O 18.0%, C⋯H/H⋯C 15.0% and N⋯H/H⋯N 10.6%) relative to other inter­molecular inter­actions. The DSC result for the enthalpy of fusion of the com­pound in its pure phase is 42.852 kJ mol^−1^, which is an indication of the thermodynamic stability of the co­crys­tal form. The co­crys­tal is thermodynamically stable, with a sum energy of −103.255 kJ mol^−1^, and the two dominating inter­actions are electrostatic and dispersion energies.

The analysis of the crystal structures in the CSD shows that the average bond distance in CONH_2_⋯N_py_ is greater than in COOH⋯N_py_. The investigation of the NH_2_⋯N_py_ synthon and inter­actions in the CSD reveals a higher occurrence of the NH_2_⋯N_py_ synthon in two similar mol­ecules and aliphatic amide–pyridyl than in aromatic amide–pyridyl systems (Chen *et al.*, 2018 ▸; Aakeröy *et al.*, 2011 ▸). Furthermore, the strength of the aromatic NH_2_⋯N_py_ bond does not correlate with the bond angles, and the strength of the NH_2_⋯N_py_ bond is observed to be influenced by the presence of close supporting inter­actions and functional groups, such as carb­oxy­lic acid and nitro groups. This suggests, amongst other things, why there are not many examples of the NH_2_⋯N_py_ synthon in the CSD.

Crystal engineers rely on the control of directional inter­actions and synthons in the assembly of functional materials (Desiraju, 2011 ▸). These strong hy­dro­gen bonds are important in the initial stage of mol­ecular aggregation; however, weak inter­actions are equally important in the mol­ecular packing at the final stage of assemblies and can induce crystal packing variations (Desiraju, 2013 ▸; Ravat *et al.*, 2015 ▸).

## Conclusions

This study conducted the synthesis of a cocrystal of nicotinamide (Nico) and 2-chloro-3-hy­droxy­pyridine (2Cl3OHPY) and analyzed the structural properties and inter­molecular inter­action of the product using both experimental and com­putational methods. The study also reveals the trends in the occurrence of the NH_2_⋯N_py_ synthon in the CSD. Understanding the structural properties and inter­molecular inter­actions in the (Nico)·(2Cl3OHPY) co­crys­tal provided in this study contributed to the mol­ecular recognition of the amide–pyridyl co­crys­tal.

The functional groups, close inter­actions and other insights generated from this study could be harnessed to potentially predict the co­crys­tallization of amide–pyridyl systems.

## Supplementary Material

Crystal structure: contains datablock(s) I, global. DOI: 10.1107/S2053229626004882/op3038sup1.cif

Structure factors: contains datablock(s) I. DOI: 10.1107/S2053229626004882/op3038Isup2.hkl

Fingerprint plots and PXRD patterns. DOI: 10.1107/S2053229626004882/op3038sup3.pdf

Supporting information file. DOI: 10.1107/S2053229626004882/op3038Isup4.cml

CCDC reference: 2433098

## Figures and Tables

**Table 1:** The average value of the hy­dro­gen-bond length of the two synthons

Synthon	Hydrogen bond	Lower quartile	Upper quartile	Average	Standard deviation
Acid–pyridyl synthon	H⋯*A* (Å)	1.68	1.84	1.77	0.19
	*D*⋯*A* (Å)	2.60	2.68	2.65	0.08
Amide–pyridyl synthon	H⋯*A* (Å)	2.14	2.36	2.26	0.18
	*D*⋯*A* (Å)	2.98	3.12	3.00	0.05

**Table 2:** Crystal structures in the CSD with aromatic amide–pyridyl synthons along their bond angles and inter­action energy (NH_2_⋯N_py_) 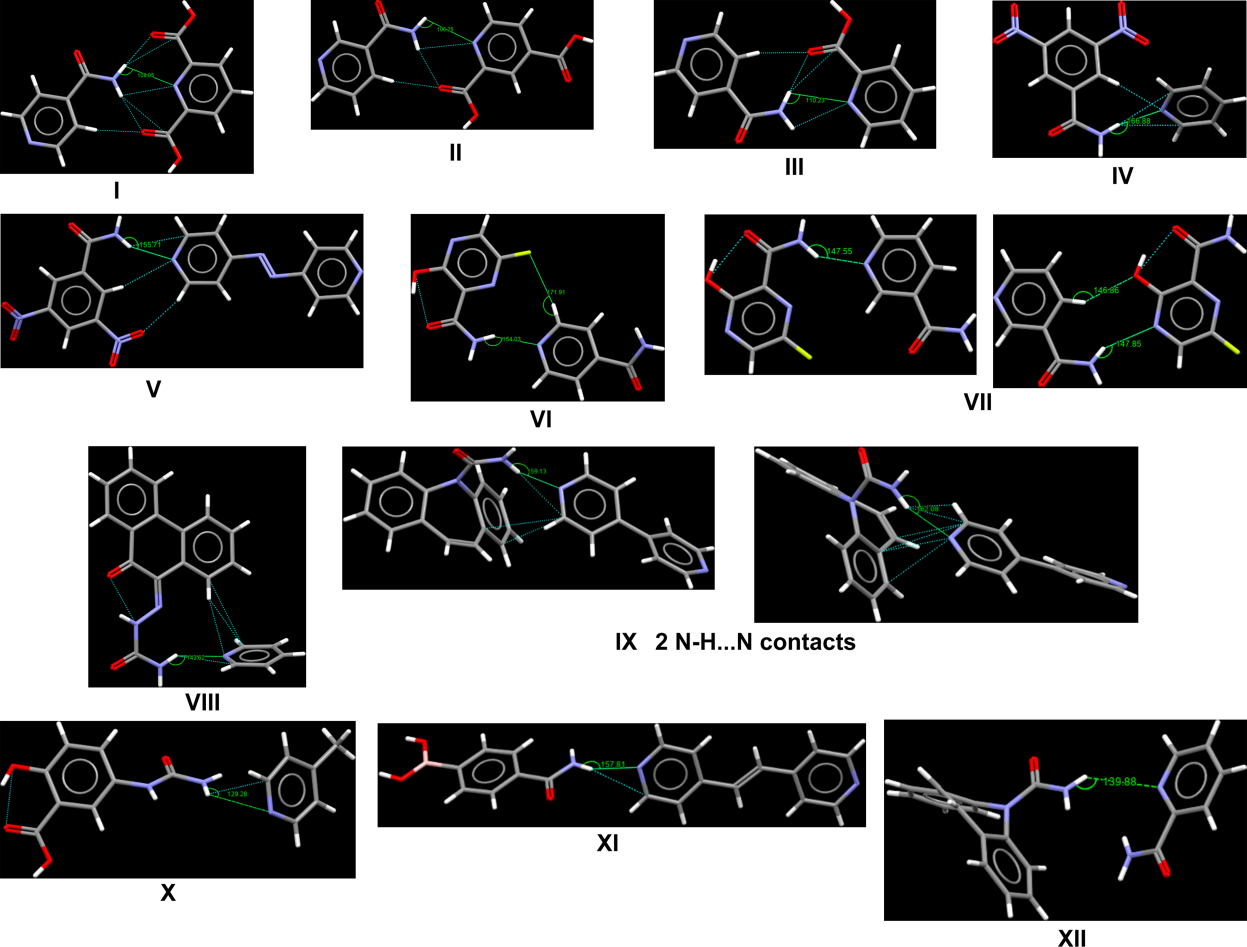

No.	CSD refcode	Reference	Bond angle (°)	Inter­action energy (kJ mol^−1^)
I	MELYEI	Aghabozorg *et al.* (2006 ▸)	108	−54.48
II	LOTTEX	Ezekiel *et al.* (2024 ▸)	106	−43.30
III	PAQMIG	Ganduri *et al.* (2017 ▸)	110	−42.84
IV	CAYJIW	Arora *et al.* (2005 ▸)	167	−47.61
V	BULJOJ	Ravat *et al.* (2015 ▸)	156	−41.84
VI	KOHPUW	Wong *et al.* (2024 ▸)	154	−33.43
VII	QOQNUJ	Li *et al.* (2024 ▸)	148	−26.48
			148	−25.19
VIII	KUVYOR	Walshe *et al.* (2015 ▸)	144	−31.34
IX	XAQQUC	McMahon *et al.* (2005 ▸)	159	−30.46
	(2 N—H⋯N contacts)		162	−34.81
X	IVORUI	Kennedy *et al.* (2016 ▸)	129	−25.15
XI	QUGRIX	Barba Hernández *et al.* (2024 ▸)	159	−24.48
XII	DOSDOI	Boycov *et al.* (2024 ▸)	140	−14.14

**Table 3:** Selected substituted pyridines for co­crys­tallization with either nicotinamide or isonicotinamide

	Substituted pyridine
Nicotinamide or	Pyridine
isonicotinamide	2-Amino­pyridine
	3-Amino­pyridine
	4-Amino­pyridine
	2-Hy­droxy­pyridine
	3-Hy­droxy­pyridine
	4-Hy­droxy­pyridine
	2-Amino-3-nitro­pyridine
	2-Amino-5-nitro­pyridine
	2-Amino-5-methyl­pyridine
	2-Amino-5-chloro­pyridine
	2-Amino-3-hy­droxy­pyridine
	2-Chloro-3-hy­droxy­pyridine
	3-Amino-2-chloro­pyridine
	4-Cyano­pyridine

**Table 4:** Experimental details

Crystal data	
Chemical formula	C_6_H_6_N_2_O·C_5_H_4_ClNO
*M*_r_	251.67
Crystal system, space group	Monoclinic, *P*2_1_/*n*
Temperature (K)	173
*a*, *b*, *c* (Å)	8.0095 (3), 6.6434 (2), 20.6585 (6)
β (°)	92.563 (1)
*V* (Å^3^)	1098.15 (6)
*Z*	4
Radiation type	Mo *K*α
μ (mm^−1^)	0.34
Crystal size (mm)	0.20 × 0.15 × 0.09

Data collection	
Diffractometer	Bruker APEXII CCD
No. of measured, independent and observed [*I* > 2σ(*I*)] reflections	19834, 2745, 2461
*R*_int_	0.041
(sin θ/λ)_max_ (Å^−1^)	0.671

Refinement	
*R*[*F*^2^ > 2σ(*F*^2^)], *wR*(*F*^2^), *S*	0.032, 0.091, 1.06
No. of reflections	2745
No. of parameters	155
H-atom treatment	H-atom parameters constrained
Δρ_max_, Δρ_min_ (e Å^−3^)	0.33, −0.23

Computer programs: *SAINT* (Bruker, 2021 ▸), *APEX4* (Bruker, 2021 ▸), *SHELXT2018* (Sheldrick, 2015*a* ▸), *SHELXL2018* (Sheldrick, 2015*b* ▸) and *OLEX2* (Dolomanov *et al.*, 2009 ▸).

**Table 5:** Hydrogen-bond geometry (Å, °)

*D*—H⋯*A*	*D*—H	H⋯*A*	*D*⋯*A*	*D*—H⋯*A*
O1—H1⋯N2	0.84	1.80	2.6323 (13)	170
N3—H3*A*⋯O2^i^	0.88	2.03	2.8883 (13)	167
N3—H3*B*⋯N1^ii^	0.88	2.37	3.2062 (14)	159

Symmetry codes: (i) 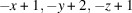; (ii) 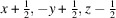.
