# Supplementary material for: Structure and intermolecular interactions of the rare amide–pyridine synthon: a cocrystal of nicotinamide and 2-chloro-3-hydroxypyridine
Source: Acta Crystallogr C Struct Chem. 2026 May 18;82(Pt 6):267–76. doi: 10.1107/S2053229626004882 (PMC13237486; doi:10.1107/S2053229626004882)
Supplement: Supplementary file 3 [file c-82-00267-sup3.pdf]

Acta Cryst C STRUCTURAL CHEMISTRY

Volume 82 (2026)

Supporting information for article:

Structure and intermolecular interactions of a rare cocrystal of nicotinamide and 2-chloro-3-hydroxypyridine

Oluwatoyin Akerele and Andreas Lemmerer

The structural and intermolecular interaction of a rare co-crystal: Nicotinamide and 2-chloro-3-hydroxypyridine (Nico-2Cl3OHPY)

Oluwatoyin Akerele and Andreas Lemmerer

Jan Boeyens Structural Chemistry Laboratory, Molecular Sciences Institute, School of Chemistry, University of the Witwatersrand, Private Bag 3, PO Wits, 2050, Johannesburg, South Africa.

Fingerprint plots (FP)

The percentage contribution of the different atoms in close contact (interaction) in the crystal structure packing of the (Nico)·(2Cl3OHPY) co-crystal is given in Fig. 7. The d_i and d_e distances—the former representing the distance from the HS to the nearest atom outside and the latter representing the distance from the HS to the nearest atom in the interior—are used to construct the FP.[67] Fig. 7 displayed the $\pi\cdots\pi$ stacking and O-H hydrogen bond interactions, as well as the split into other individual elements.

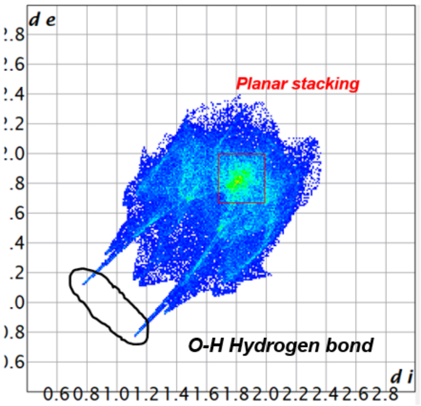

Figure 7: Finger plot of (Nico)·(2Cl3OHPY) co-crystal, showing non-covalent interactions

The interactions between H···H account for the largest portion of the HS region in the 2D fingerprint maps, representing 24.5%. The interactions between O···H/H···O account for 18.0% of the six sharp spikes, whereas Cl···H/H···Cl and N···H/H···N interactions account for 16.2% and 10.6%, respectively. The other inter-contacts are C···H/H···C (15.0%), C···C (5.7%), N···C/C···N (4.2%), O···C/C···O (2.4%), Cl···C/C···Cl (2.0%), Cl···O/O···Cl (1.2%), Cl···N/N···Cl (0.1%), and O···N/N···O (0.1%), as shown in Fig. 8.

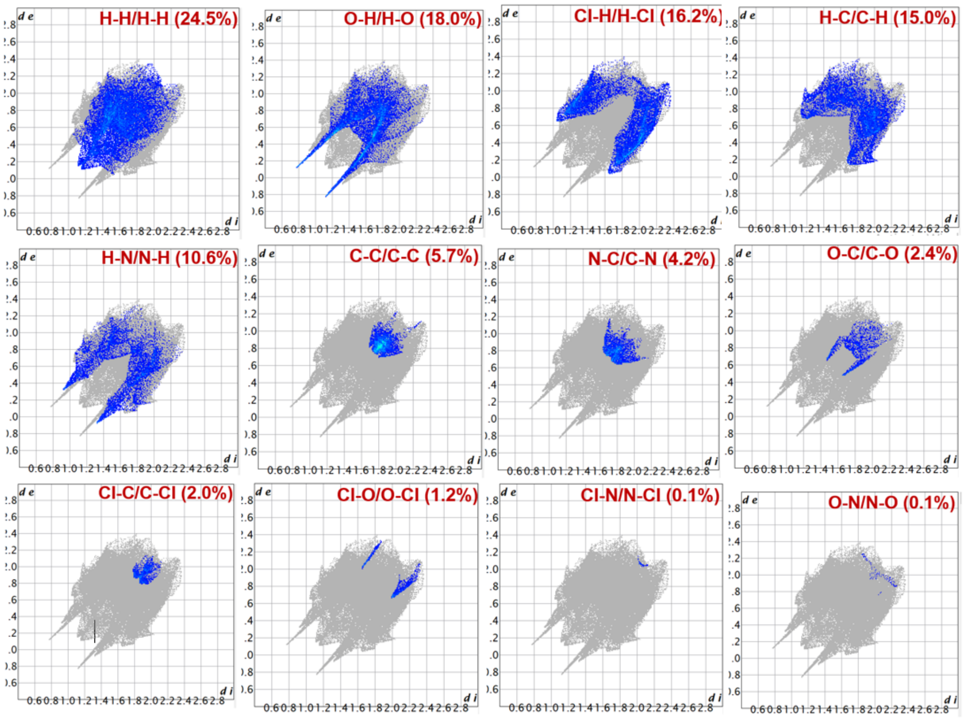

Figure 8: 2D fingerprint plots of the (Nico)·(2Cl3OHPY) structure

Surfaces: d_{norm}, curvedness, and shape index

The color-coded distances representing the different intermolecular interactions of the structures were mapped onto the Hirshfeld surfaces.

a. The HS is plotted over the d_{norm} in Fig. 9. The intensity of red spots is a qualitative indicator of the contacts' strength, as seen in the intermolecular interactions presented. The NH···O hydrogen bonds produce two intense red patches (Fig. 9a), which is in correlation with the strength of energy for the dimer hydrogen bond in Fig. 5b. The C-O and H-H contacts are indicated by a light red region (Fig. 9b).

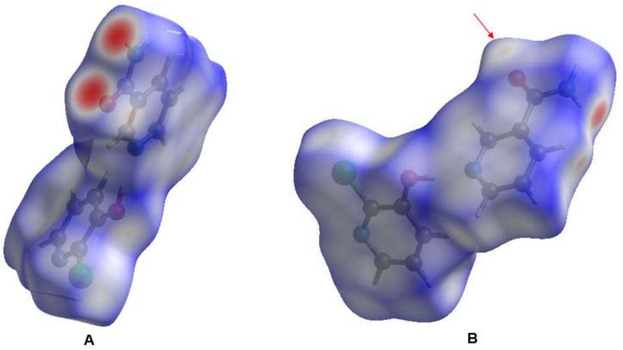

Figure 9: Three-dimensional Hirshfeld surface of the (Nico)·(2Cl3OHPY) structure mapped on d_{norm} between -0.5487 and 1.0648 a.u (a) View along the *b*-axis (a) *a*-axis

b. A planar surface area is shown by green flat patches. The curved surfaces of the (Nico)·(2Cl3OHPY) structure in Fig. 10 display a flat surface region indicative of the planar stacking interaction between molecules.

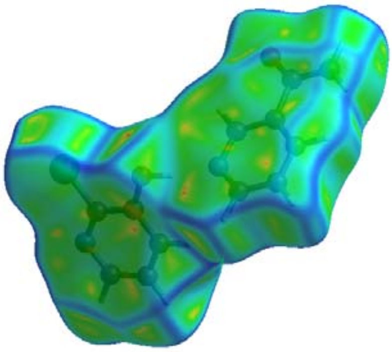

Figure 10: Three-dimensional Hirshfeld surface view along the *a*-axis of (Nico)·(2Cl3OHPY) mapped on curvedness between -4.0000 and 0.4000 a.u.

c. The presence of red and blue triangles on the shape-indexed surface of the (Nico)·(2Cl3OHPY) (Fig. 11) clearly shows the presence of π···π stacking interactions in the structure of the Nico-2Cl3OHPY co-crystal.[66,78-70] This result is consistent with the π···π stacking interactions observed in the crystal packing of (Nico)·(2Cl3OHPY) in Fig. 6.

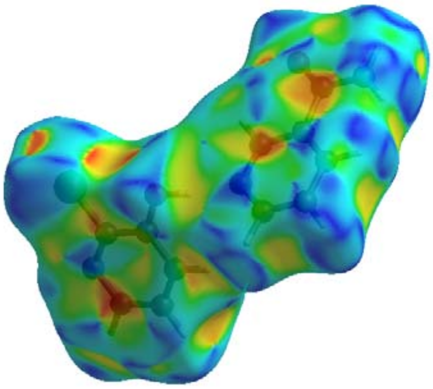

Figure 11: Three-dimensional Hirshfeld surface of (Nico)·(2Cl3OHPY) mapped on shape index between -1.0000 and 1.0000 a.u. view along the a-axis.

Powder X-Ray Diffraction (PXRD)

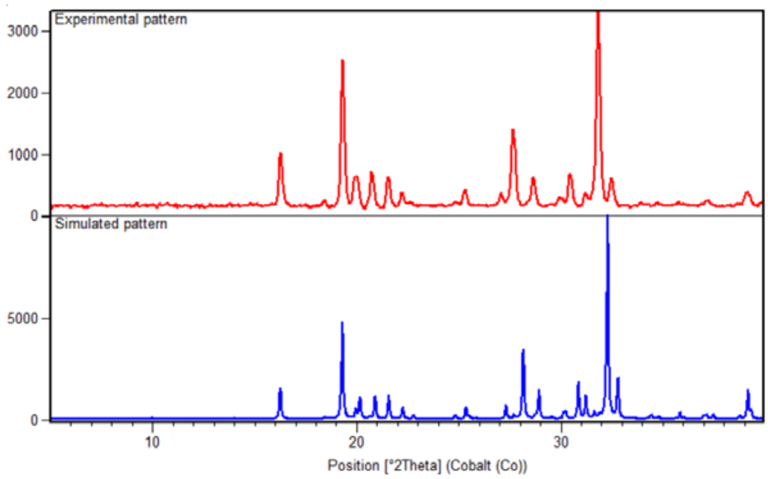

Figure SI1: Overlay of powder pattern of the co-crystal, red is the experimental patterns obtain with a Co X-ray source (λ = 1.78896 Å) and blue is simulated pattern.

Mechanochemical Results_PXRD

Isonicotinamide

The powder pattern result of isonicotinamide:pyridine and isonicotinamide:3-amino-2-chloro-pyridine were not included because they resulted into liquid.

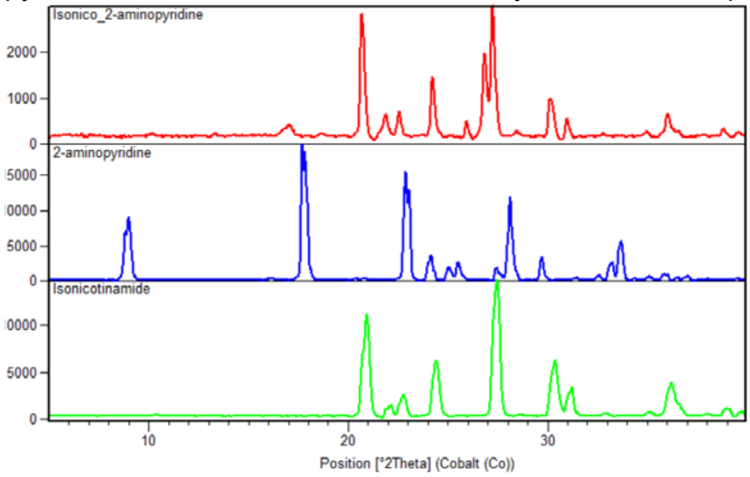

Figure S2: Overlay of parent compounds and resulting multi-component; red is supposed co-crystal, blue is 2-aminopyridine, and green is the isonicotinamide.

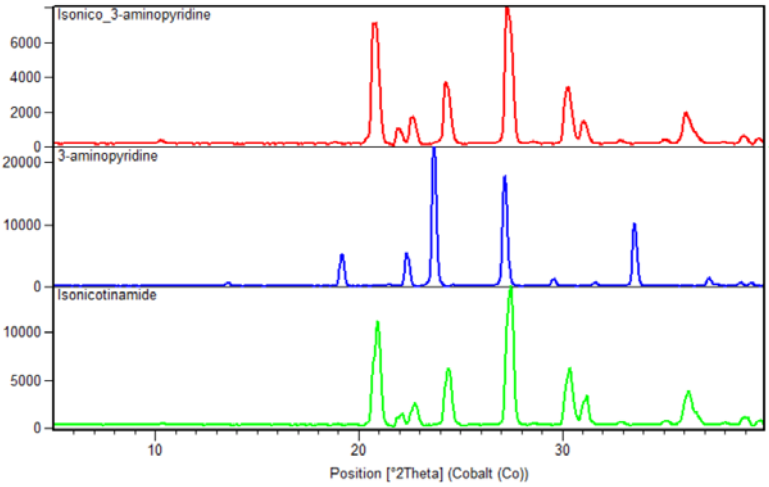

Figure SI3: Overlay of parent compounds and resulting multi-component; red is supposed co-crystal, blue is 3-aminopyridine, and green is the isonicotinamide.

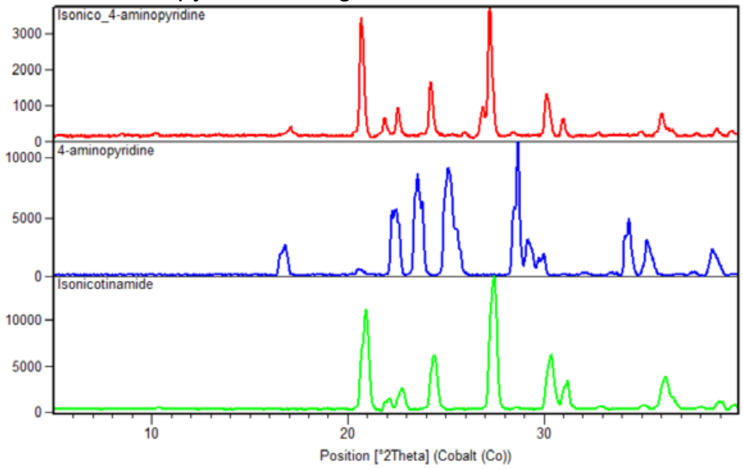

Figure SI4: Overlay of parent compounds and resulting multi-component; red is supposed co-crystal, blue is 4-aminopyridine, and green is the isonicotinamide.

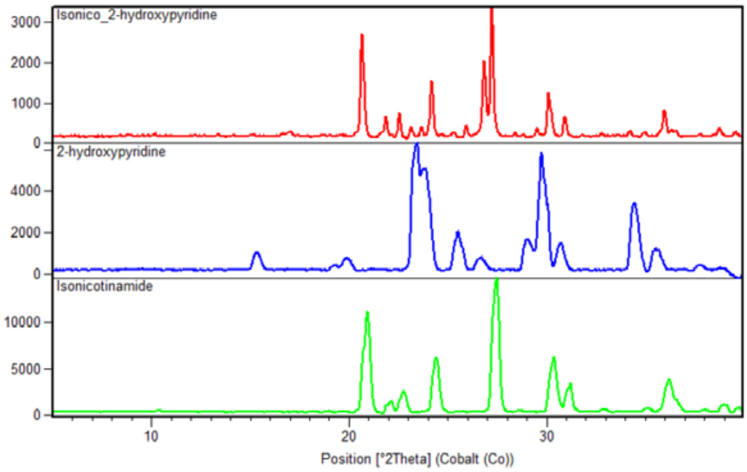

Figure SI5: Overlay of parent compounds and resulting multi-component; red is supposed co-crystal, blue is 2-hydroxypyridine, and green is the isonicotinamide.

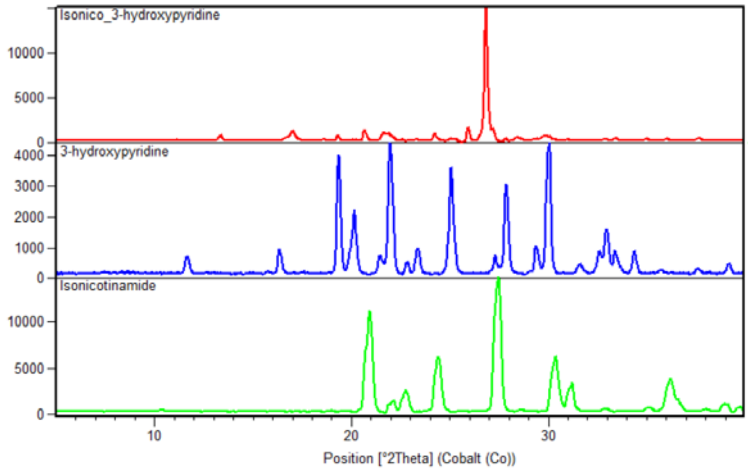

Figure SI6: Overlay of parent compounds and resulting multi-component; red is supposed co-crystal, blue is 3-hydroxypyridine, and green is the isonicotinamide.

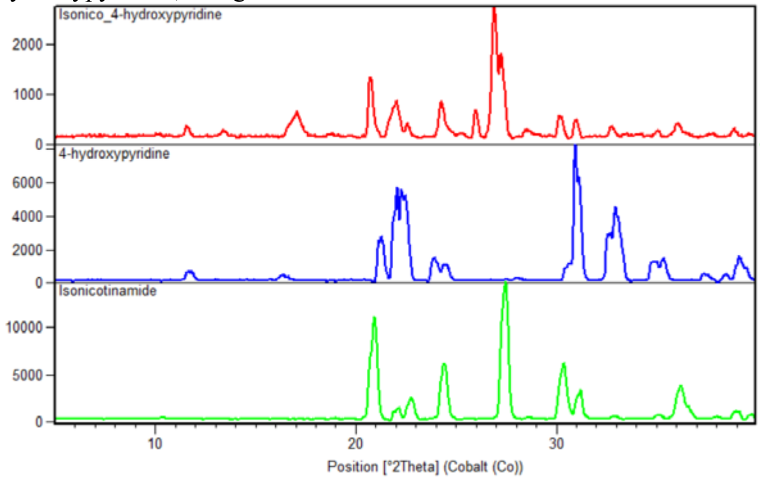

Figure SI7: Overlay of parent compounds and resulting multi-component; red is supposed co-crystal, blue is 4-hydroxypyridine, and green is the isonicotinamide.

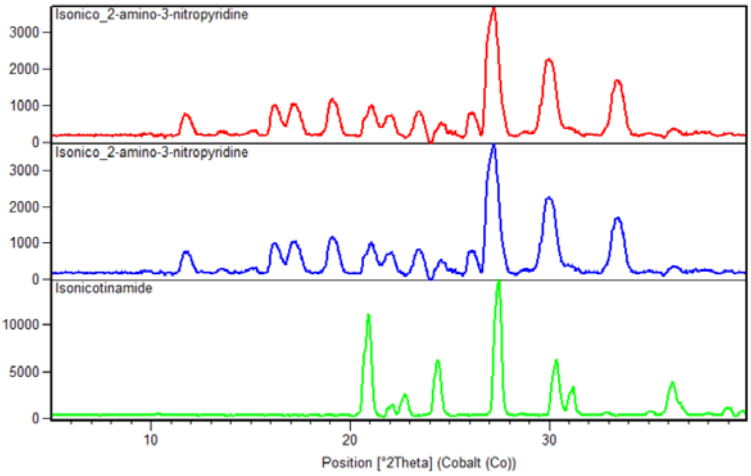

Figure SI8: Overlay of parent compounds and resulting multi-component; red is supposed co-crystal, blue is 2-amino-3-nitropyridine, and green is the isonicotinamide.

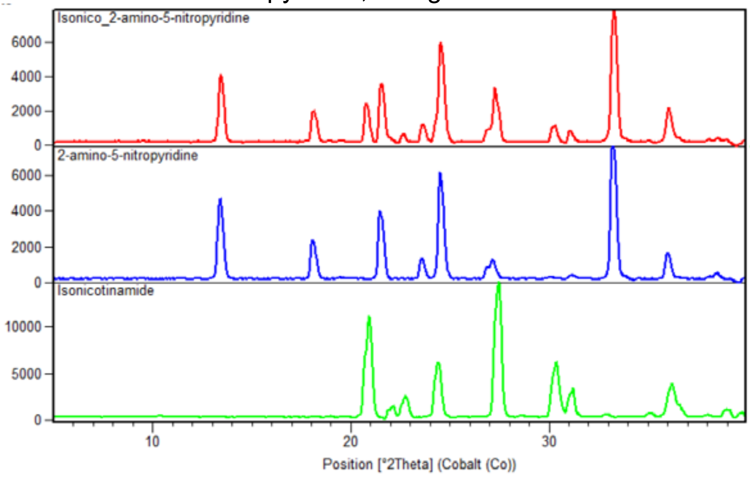

Figure SI9: Overlay of parent compounds and resulting multi-component; red is supposed co-crystal, blue is 2-amino-5-nitropyridine, and green is the isonicotinamide.

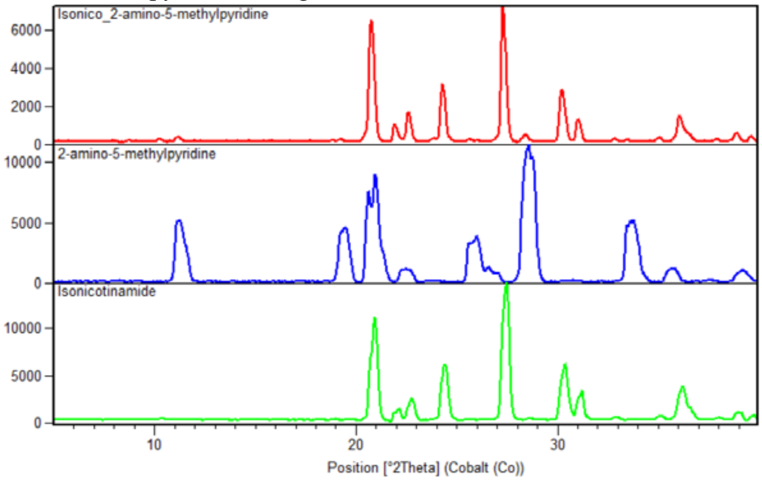

Figure SI10: Overlay of parent compounds and resulting multi-component; red is supposed co-crystal, blue is 2-amino-5-methylpyridine, and green is the isonicotinamide.

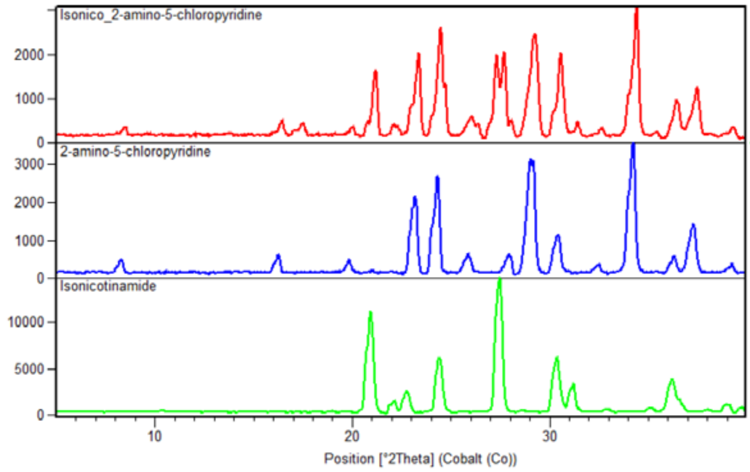

Figure SI11: Overlay of parent compounds and resulting multi-component; red is supposed co-crystal, blue is 2-amino-5-chloropyridine, and green is the isonicotinamide.

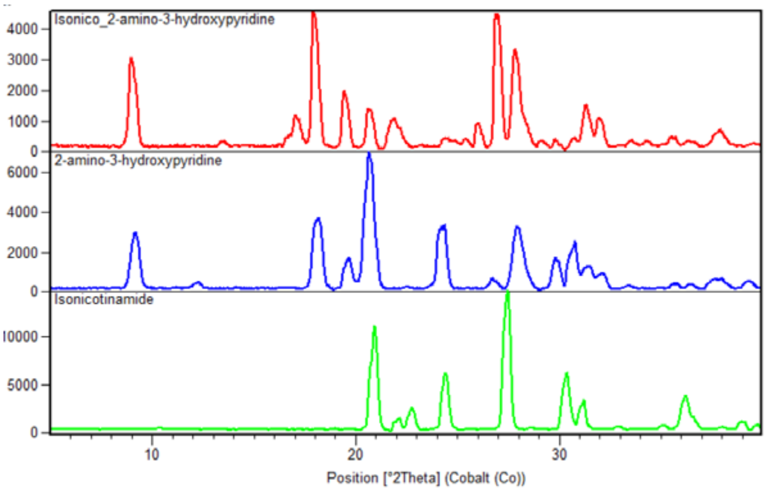

Figure SI12: Overlay of parent compounds and resulting multi-component; red is supposed co-crystal, blue is 2-amino-3-hydroxypyridine, and green is the isonicotinamide.

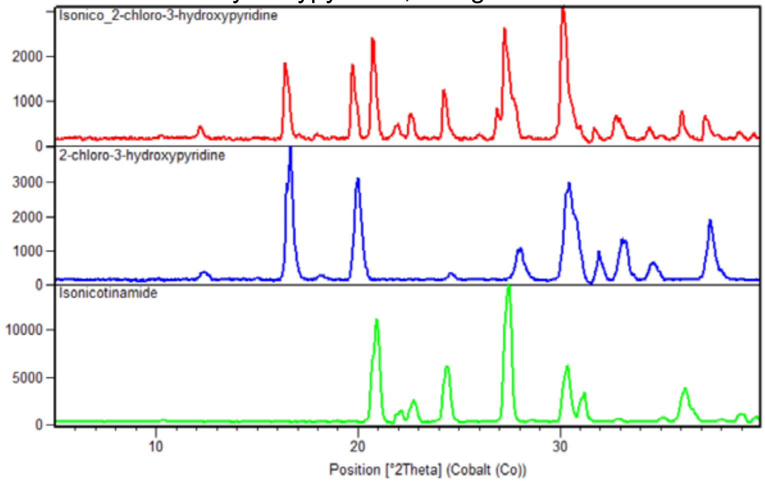

Figure SI13: Overlay of parent compounds and resulting multi-component; red is supposed co-crystal, blue is 2-chloro-3-hydroxypyridine, and green is the isonicotinamide.

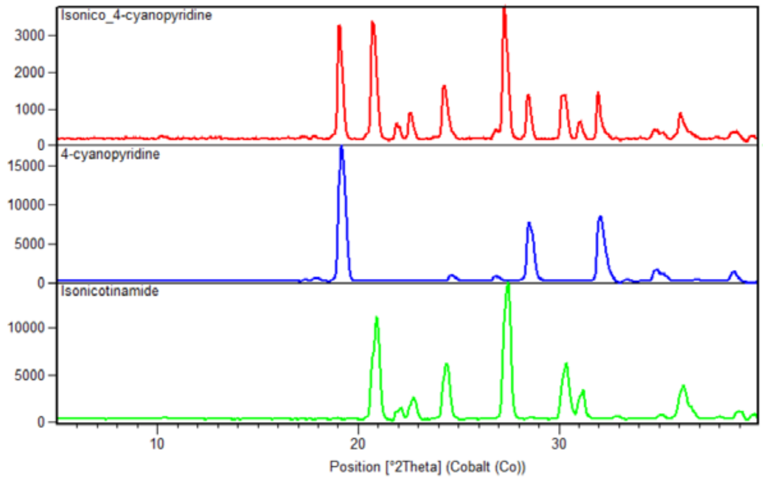

Figure SI14: Overlay of parent compounds and resulting multi-component; red is supposed co-crystal, blue is 4-cyanopyridine, and green is the isonicotinamide.

Nicotinamide

The powder pattern result of nicotinamide-pyridine and nicotinamide-4-cyanopyridine were not included because they resulted into liquid. The powder pattern of the novel co-crystal (Nicotinamide:2-chloro-3-hydroxypyridine) is in the manuscript, see Fig. 2.

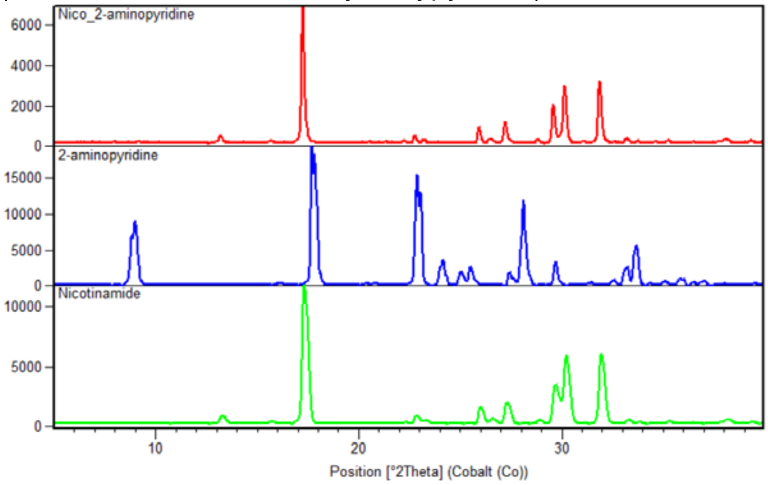

Figure SI15: Overlay of parent compounds and resulting multi-component; red is supposed co-crystal, blue is 2-aminopyridine, and green is the Nicotinamide.

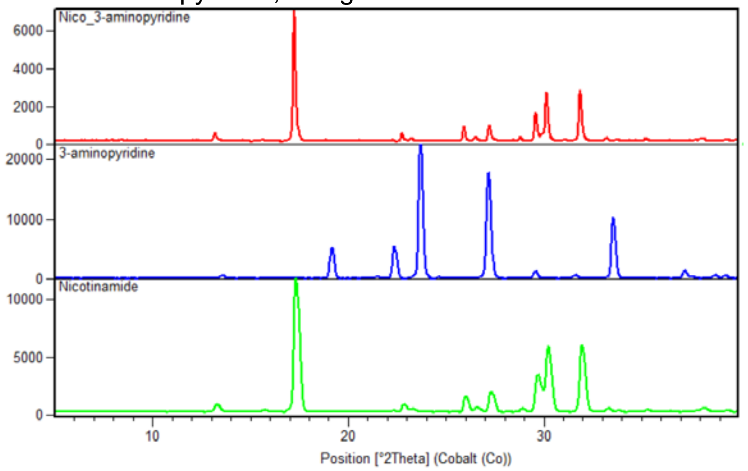

Figure SI16: Overlay of parent compounds and resulting multi-component; red is supposed co-crystal, blue is 3-aminopyridine, and green is the Nicotinamide.

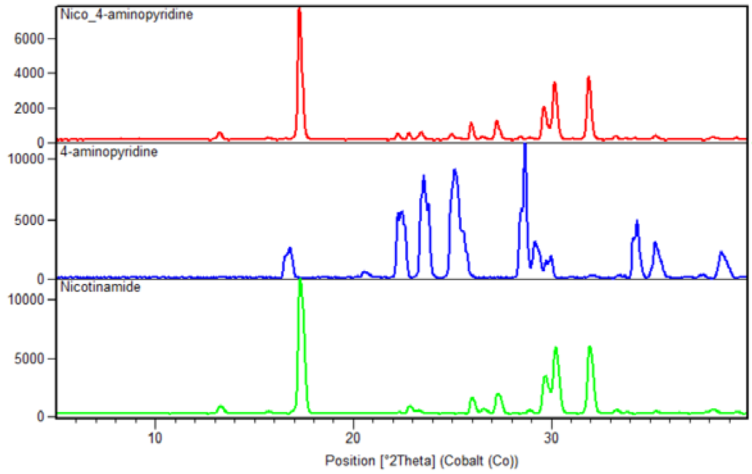

Figure SI17: Overlay of parent compounds and resulting multi-component; red is supposed co-crystal, blue is 4-aminopyridine, and green is the Nicotinamide.

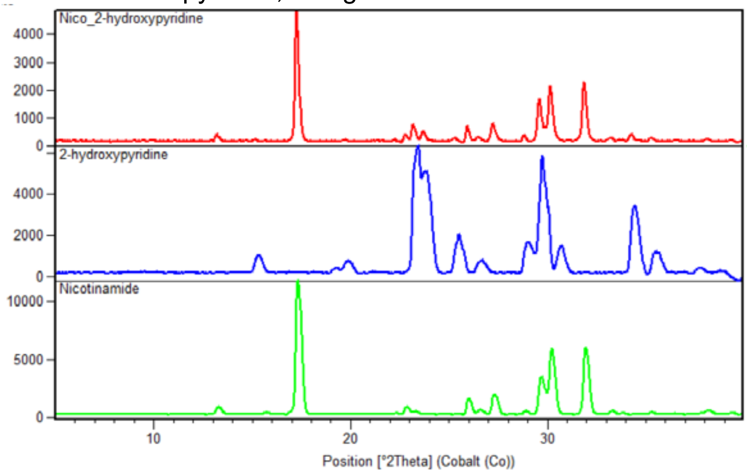

Figure SI18: Overlay of parent compounds and resulting multi-component; red is supposed co-crystal, blue is 2-hydroxypyridine, and green is the Nicotinamide.

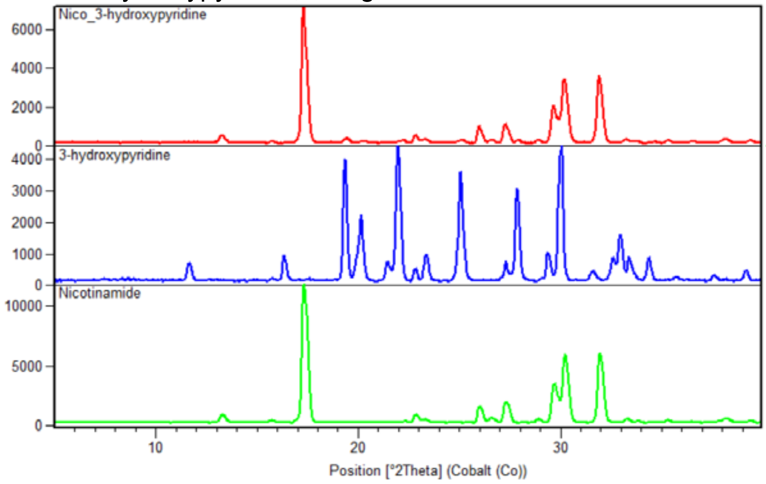

Figure SI19: Overlay of parent compounds and resulting multi-component; red is supposed co-crystal, blue is 3-hydroxypyridine, and green is the Nicotinamide.

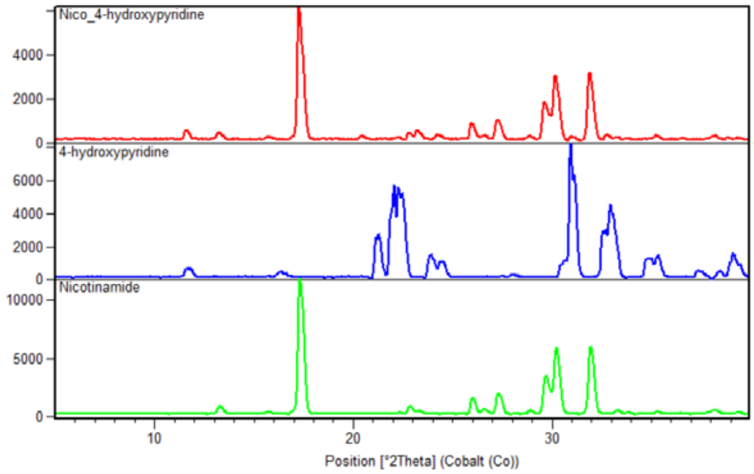

Figure SI20: Overlay of parent compounds and resulting multi-component; red is supposed co-crystal, blue is 4-hydroxypyridine, and green is the Nicotinamide.

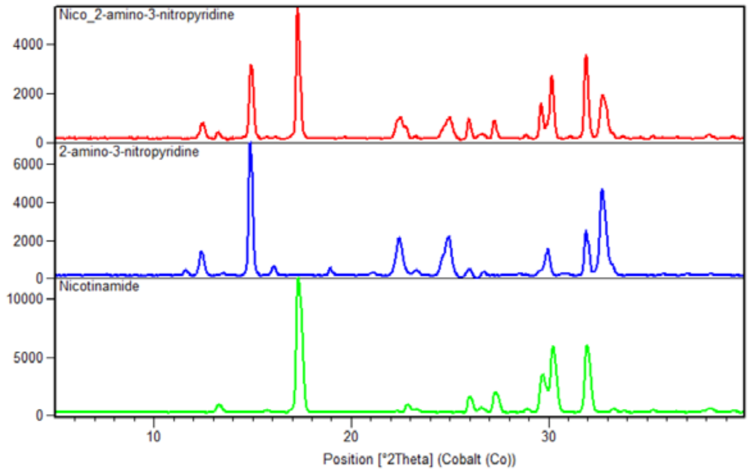

Figure SI21: Overlay of parent compounds and resulting multi-component; red is supposed co-crystal, blue is 2-amino-3-nitropyridine, and green is the Nicotinamide.

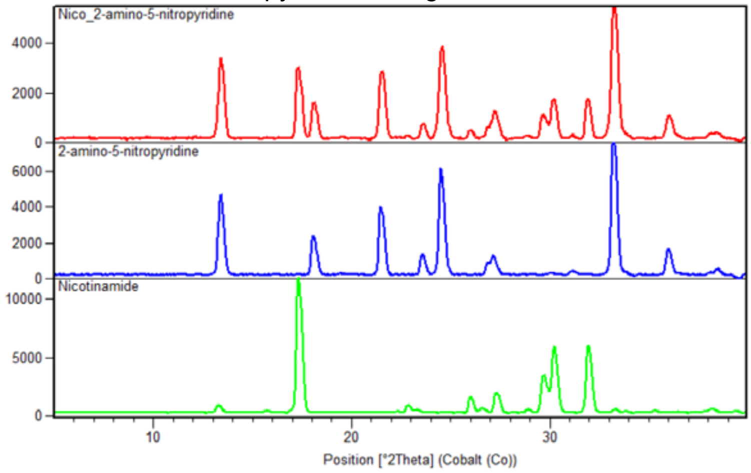

Figure SI22: Overlay of parent compounds and resulting multi-component; red is supposed co-crystal, blue is 2-amino-5-nitropyridine, and green is the Nicotinamide.

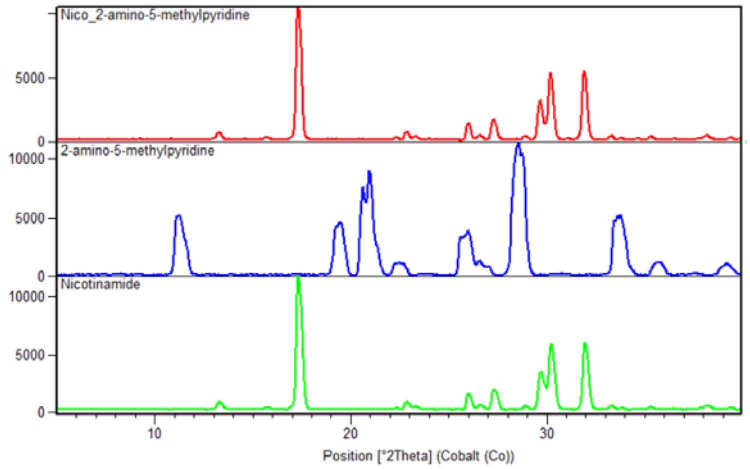

Figure SI23: Overlay of parent compounds and resulting multi-component; red is supposed co-crystal, blue is 2-amino-5-methylpyridine, and green is the Nicotinamide.

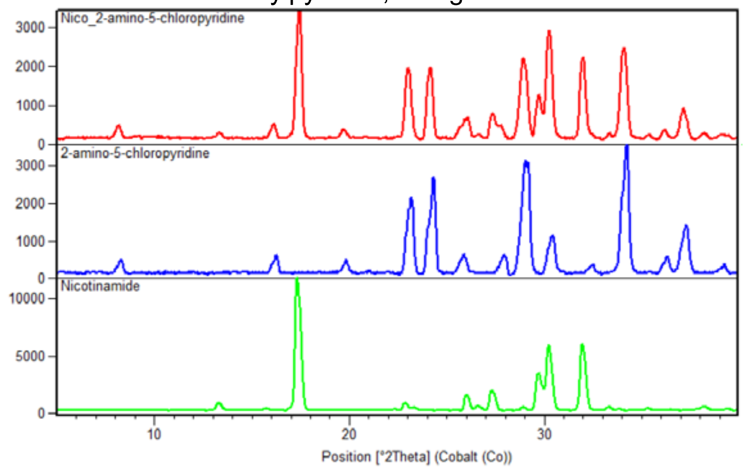

Figure SI24: Overlay of parent compounds and resulting multi-component; red is supposed co-crystal, blue is 2-amino-5-chloropyridine, and green is the Nicotinamide.

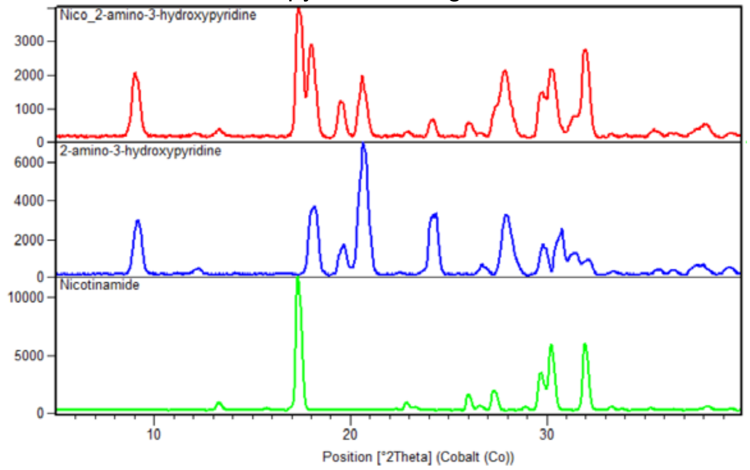

Figure SI25: Overlay of parent compounds and resulting multi-component; red is supposed co-crystal, blue is 2-amino-3-hydroxypyridine, and green is the Nicotinamide.

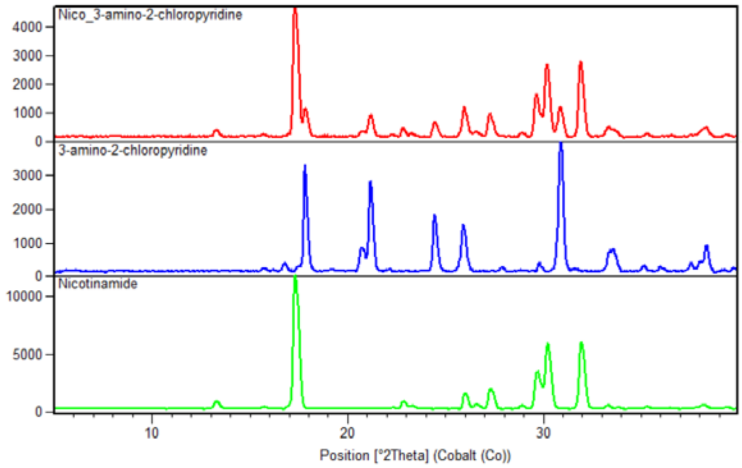

Figure SI26: Overlay of parent compounds and resulting multi-component; red is supposed co-crystal, blue is 3-amino-2-chloropyridine, and green is the Nicotinamide.

Crystal explorer results

Interaction Energy

-81.442 (E_{ele}), -18.963 (E_{pol}), -74.0132(E_{dis}), 71.1627 (E_{rep}), and -103.255 (E_{tot})

Lattice Energy

-66.750 (E_{ele}), -14.097 (E_{pol}), -64.650 (E_{dis}), 58.138 (E_{rep}), and -87.358 (E_{tot})
